# Utilization of full postnatal care services among rural Myanmar women and its determinants: a cross-sectional study

**DOI:** 10.12688/f1000research.15561.1

**Published:** 2018-08-01

**Authors:** Aye Sandar Mon, Myo Kyi Phyu, Wilaiphorn Thinkhamrop, Bandit Thinkhamrop

**Affiliations:** 1 Doctor of Philosophy in Epidemiology and Biostatistics Program, Faculty of Public Health, Khon Kaen University, Mueang Khon Kaen, Khon Kaen, 40002, Thailand; 2 Department of Biostatistics, University of Public Health, Yangon, 11131, Myanmar; 3 Department of Preventive and Social Medicine, University of Medicine (2), Yangon, Yangon, 11031, Myanmar; 4 Doctor of Public Health Program, Data Management and Statistical Analysis Center, Faculty of Public Health, Khon Kaen University, Mueang Khon Kaen, Khon Kaen, 40002, Thailand; 5 Department of Epidemiology and Biostatistics, Data Management and Statistical Analysis Center, Faculty of Public Health, Khon Kaen University, Mueang Khon Kaen, Khon Kaen, 40002, Thailand

**Keywords:** postnatal care, full PNC utilization, rural women, Myanmar

## Abstract

**Background: **Mothers and their newborns are vulnerable to threats to their health and survival during the postnatal period. Full postnatal care (PNC) uptake decreases maternal deaths and is also essential for first 1,000 days of newborn’s life, but PNC usage is usually inadequate in rural areas. Little is known about the full PNC utilization among rural Myanmar women. This study, therefore, aimed to study the situation of the utilization of full PNC and examine its determinants.

**Methods: **This community-based cross-sectional study was conducted in selected villages of the Magway Region, Myanmar. A total of 500 married women who had children aged under 2 years were selected using multistage cluster sampling and interviewed with semi-structured questionnaires. The determinants of full PNC usage were identified by generalized estimating equation (GEE) under a logistic regression framework.

**Results: **Among 500 rural women, around a quarter (25.20%; 95% confidence interval (CI), 21.58-29.21%) utilized full PNC. Multivariable analysis revealed that factors associated with full PNC usage included mothers attaining educational level of secondary or higher (adjusted odds ratio (AOR), 2.16; 95% CI, 1.18-3.94), belonging to higher income level (AOR, 2.02; 95% CI, 1.11-3.68), having male involvement (AOR, 2.19; 95% CI, 1.02-4.69), being of low birth order (i.e. the first birth) (AOR, 3.26; 95% CI, 1.80-5.91), and having awareness of postnatal danger signs (AOR, 2.10; 95% CI, 1.15-3.83). Moreover, the presence of misconceptions on postnatal practice was identified as a strong barrier to adequate PNC usage (AOR, 0.12; 95% CI, 0.04-0.36).

**Conclusion: **Most of the rural women practiced inadequate PNC in Myanmar. Maternal healthcare services at rural areas should be intensively promoted, particularly among women who had high birth order (greater number of births). Health education regarding perinatal misconceptions and danger signs, and benefits of full PNC services usage should be emphasized and urgently extended.

## Introduction

In Myanmar, eliminating preventable maternal mortality remains one of the critical challenges to the health system, despite the fact that maternal and child health care has been prioritized. The maternal mortality ratio (MMR) was estimated as 282 per 100,000 live births (LBs) in 2014 Myanmar census report ^1^. In South-East Asian region, Myanmar has a higher MMR than the regional average, which is 140 per 100,000 LBs ^1^. The leading cause of maternal death was post-partum haemorrhage (PPH), and the second and third-leading causes were pregnancy-induced hypertension and abortion, respectively. Over three-quarters (77.4%) of maternal deaths in Myanmar occurred in women who resided in rural areas ^2^. Even though rural women are likely to have higher birth rates, most of them have greater reluctance in seeking, reaching and receiving care from skilled providers ^3^.

Increasing the quality and skilled postnatal care has recently been highlighted as a method of reducing preventable maternal mortality ^4^. Moreover, effective and adequate PNC is also essential for the first 1,000 days of a child’s life. Improving the health of pregnant women and new mothers will not only reduce maternal morbidity and mortality, but also further reduce child mortality. It has been shown that motherless children have a higher chance of dying before their second birthday than those who have mothers alive ^5^. The highest risk of maternal mortality is during delivery and in the immediate postnatal period, especially the first 24 hours ^6^. Therefore, the World Health Organization (WHO) recommended the optimal timing of PNC should start as early as possible within 24 hours after birth, even if birth occurs at home. The recommended numbers of postnatal visits are at least three additional post natal contacts, in addition to the first contact within 24 hours of birth: on day 3, between days 7 and 14, and 6 weeks postpartum ^7^.

The postpartum period is defined as the first six weeks (42 days) after birth. This period poses substantial risks and hazards for maternal and neonatal health, and a lack of quality health care during this time may result in mortality or disability, in addition to missed opportunities to promote healthy behaviors. The first hours and days after birth are the most crucial for both mother and neonate, despite the fact that those in the postnatal period are paid less attention by skilled care providers compared to those in the antenatal and intranatal periods ^7, 8^.

A number of international studies have been conducted to determine the factors associated with postnatal care utilization in developing countries. Some have emphasized the timing of postnatal care visits ^9, 10^, but others have considered whether women received PNC at least once, regardless of the timing of the first visit or the number of visits ^11– 15^. In Myanmar, literature concerning postnatal care remains limited in spite of having many studies about antenatal and intranatal care. This study aims to explore the magnitude of rural women who received full PNC in addition to push and pull factors for full PNC utilization in Myanmar.

## Methods

This community-based cross-sectional survey was conducted at selected villages (anonymized for ethical reasons) in the Magway Region, which was chosen because its MMR, 343.6 per 100,000 LBs ^1^, is higher than union average (282 per 100,000 LBs) and then 85% of residents in this region are from rural populations ^16^. Data were collected between November 2016 and January 2017.

### Study participants

The required sample size of 500 participants was estimated based on the multiple logistic regression analysis, as described previously ^17^. Married women aged 15-49 years who had children aged under 2 years of age and provided informed consent were eligible for this study. Woman who could not communicate properly due to physical or mental ill health were excluded from this study. The eligible samples were obtained by applying multistage cluster sampling method. Firstly, out of 26 townships, 4 were selected by simple random sampling using a lottery. From each township, a random selection of 5 villages (having not less than 18 women who had delivered 2 years prior to the survey) was done. As a result, 500 women fitting the eligibility criteria were recruited in person, with the assistance of local health authorities, from 21 villages by cluster sampling. The 2-year recall period was used to minimize recall bias.

### Data collection

Data were collected by face to face interview using semi-structured questionnaires ( Supplementary File 1). Reliability of 0.86 was estimated by using Cronbach’s alpha. Validity was arranged by the three experts to obtain the finalized version of questionnaire. Preceding the interview, the researcher trained 10 enumerators for data collection and also explained the objectives and facts to follow while asking the questionnaires. Data were collected after participants had been informed about the purpose of the study, ensuring confidentiality to those taking part in the study.

### Assessment of variables

In this study, the outcome variable was utilization of full PNC which was defined as the participants receiving at least four postnatal visits and the first visit within 24 hours of delivery. For analysis, the outcome responses were dichotomized into the women who reported less than four postnatal visits or postnatal care after 24 hours =0 and those who received four or more postnatal visits and the first visit within 24 hours =1. The independent variables measured were as followings: socio-demographic variables such as age of respondents, education level, average monthly per-capita income, male involvement, accessibility to PNC services. Moreover, knowledge of postnatal danger signs and perception on traditional birth attendants (TBAs) were also defined as explanatory variables. Finally, birth order (i.e. the order that the child was born to his/her family), number of AN visits and misconception regarding postnatal practices were considered as important independent variables in this study.

Some independent variables are explained in detail as follows. Male involvement was considered if the woman was provided with transportation assistance for perinatal visits by her husband and the couple had mutual discussion for maternal healthcare usage. Accessibility to maternal care was defined as a combination of the time spent for travelling to the nearest health center and whether the mother could visit there during any season; that is, if the nearest health center was situated within less than 2 hours travelling distance and could be visited during any season, especially rainy season, this was counted as easy accessibility to nearest health center, otherwise, as difficulty in access. Regarding misconceptions, if a woman avoided certain foods, had behavioral restrictions or customs/practices that might threaten the health and survival of mothers and their babies within postnatal period, she belonged to the category of women having misconception on postnatal practices. The outcome variable and most of the independent variables were measured as categorical ones, except age, family income, birth order, numbers of antenatal visits and postnatal visits. However, for more simple analysis and better interpretation purposes, all numerical independent variables were categorized.

### Data analysis

The statistical analysis was conducted using the STATA version 13.1. The socio-demographic and background characteristics of respondents were presented as frequencies and percentages for categorical variables and as summary statistics, such as mean ± standard deviation for continuous variables. The full PNC utilization rate with 95% confidence interval (CI) was also described. To explore the determinants on full PNC utilization, odds ratio with 95% CI was estimated using a generalized estimating equation (GEE) under multiple logistic regression framework. To take into account the correlation of an event occurring within the same village (i.e. those in the same village having similar access to a health clinic), for estimation of standard error, the GEE was applied ^18^. The factors which were significant at p-value less than 0.25 in bivariate analysis were included in the GEE method. All statistical tests were two-sided and p-values less than 0.05 were considered as statistically significant.

### Ethical consideration

The Khon Kaen University Ethics Committee for human research with reference number [HE592256] and the Ethical Committee of University of Public Health, Yangon, Myanmar [Ethical (6/2016)] approved this study. Permission to conduct this study was obtained from local responsible persons and health authorities (i.e. village administrative authorities and health authorities from Magway Regional Public Health Department, respectively). Participation in this study was entirely voluntary and informed consent was taken from all participants prior to interview. For participants younger than 18 years, consent was obtained from the individual’s guardian.

## Results

### Background characteristics of the respondents

Out of 500 respondents, nearly half of them (48.2%) were in the young adult age group of 25 to 35 years. The participants were aged between 17 and 47 years, with a mean age of 29.72±6.6 years ( Table 1). Majority of the respondents and their spouses were in primary or below level of education, accounting 72.2% and 63.8% respectively. About 64% of the interviewee had no more than five family members. More than half of the respondents (60.8%) had low incomes (less than 50,000 Myanmar kyats (MMK)). Regarding accessibility, about half of respondents (44.8%) encountered difficulty in accessing their nearest health center (that is, they experienced more than 2 hours travel there or it was not easily accessed in the rainy season). In connection with male involvement, 46.8% of the participants were provided with assistance from their husband regarding maternal care usage, such as transportation assistance, and mutual discussion for seeking and receiving maternal healthcare services.

**Table 1.** Background characteristics of the participants (n=500).

Characteristics		Number	Percent
Age	<25 years	125	25.0
	25–35 years	241	48.2
	>35 years	134	26.8
Education attainment	Primary or below level	361	72.2
	Secondary or higher level	139	27.8
Husband’s education attainment	Primary or below level	319	63.8
	Secondary or higher level	181	36.2
Family size	<5 members	323	64.6
	≥5 members	177	35.4
Per capita income	<50,000 MMK	304	60.8
	≥50,000 MMK	196	39.2
Access to nearest health center	Not easy	224	44.8
	Easy	276	55.2
Male involvement	No	266	53.2
	Yes	234	46.8

### Factors relating to maternal healthcare received during last child delivery

The average number of children that the respondents had during the study period was 2 (SD=1.4) and 34 respondents (6.8%) had 5 children and more. For just under half of the mothers (47.6%), the last child recently delivered was their first born ( Table 2). Most of the mothers (76.2%) had low awareness of postnatal danger signs, including neonatal health risks. On the other hand, around a quarter (23.8%), classified as having a high level of awareness of postnatal danger signs, could name at least 3 out of 8 postpartum danger signs and 1 out of 6 neonatal danger signs. Nearly 50% perceived TBAs as skilled care givers. Only half of mothers received maternal healthcare (antenatal, intranatal and postnatal) from skilled healthcare providers, who included doctors, nurses, lady health visitors (skilled maternal care providers in rural areas) and midwives. Slightly under a quarter (23.6%) did not take antenatal care at all. Nearly two-thirds of the women in the study (64.4%) selected their home as their place of delivery. About one-third of mothers (32.6%) did not take postnatal care and just over a quarter (27.2%) received at least 4 visits (the WHO-recommended number of visits). Among the 337 respondents who took postnatal care, 83.68% received their first postnatal contact with skilled provider within 24 hours of delivery (the WHO-recommended timing of the first visit). The majority of these individuals (about 90%) received health services for both mother (84.8%) and newborns (97.3%). Regarding receipt of health education on breastfeeding and postnatal danger signs, around half of the mothers were provided with this information (breastfeeding, 48.1%; postnatal danger signs, 51.6%). Moreover, just under half of mothers could get knowledge about contraception methods (49.3%) although over three-quarters of them (75.4%) were provided with contraceptives. Almost all of them (98.8%) were given postnatal supplements, such as vitamin B1 and iron. Out of the 500 women, almost half of them (49.6%) had misconception regarding postnatal practices; these included food taboos such as avoiding the consumption of meat and some vegetables or behavioral restrictions such as avoiding going outside the delivery room within 7 days of the birth and massaging lower abdomen for the removal of impure blood.

**Table 2.** Factors related to maternal healthcare received during last child delivery.

Characteristics		Number	Percentage
Birth order	Second or higher	262	52.4
	First	238	47.6
Awareness of postnatal danger signs	Low level	381	76.2
	High level	119	23.8
Acceptance of TBA	Not accepted	256	51.2
	Accepted	244	48.8
Type of maternal care provider	Non-skilled	227	45.4
	Skilled	273	54.6
ANC visits	No visit	118	23.6
	<4 visits	120	24.0
	≥4 visits	262	52.4
Place of delivery	Home	322	64.4
	Health facility	178	35.6
PNC visits	No PNC	163	32.6
	<4 visits	201	40.2
	≥4 visits	136	27.2
Timing of PNC (n=337)	First 24 hours	282	83.7
	24–48 hours	3	0.9
	48–72 hours	19	5.6
	3–7 days	7	2.1
	>7 days	26	7.7
Receipt of PNC services * (n=337)	Maternal checkup	286	84.8
	Neonatal checkup	328	97.3
	HE on breastfeeding	162	48.1
	HE on postnatal danger signs	174	51.6
	HE on contraceptives	166	49.3
	Provision of contraceptives	254	75.4
	Provision of supplements	333	98.8
Postnatal complication	No	480	96.0
	Yes	20	4.0
Postnatal food restriction	No	267	53.4
	Yes	233	46.6
Postnatal behavioral restriction	No	258	51.6
	Yes	242	48.4
Misconception regarding postnatal practice	No	252	50.4
	Yes	248	49.6

*Those that received more than one PNC service. TBA, traditional birth attendant.

### Determinants of full PNC utilization: Bivariate analysis

Of the 500 women in this study with children under 2 years age, 126 utilized full PNC, i.e. they received at least four postnatal visits and their first visit within 24 hours after childbirth (25.20% (95%CI, 21.58-29.21)) ( Table 3). The results from bivariate analysis presented as the crude odds ratio (OR) along with its 95% CI, and P-value of each variable revealed that all of the factors in the Table 3 were statistically significant associated with full PNC: these were composed of age, education attainment of respondents and their husbands, income, accessing to health center, male involvement, birth order, awareness of postnatal danger signs, acceptance of TBA, types of health care provider, number of AN visits, place of delivery and misconception regarding postnatal practices.

**Table 3.** Utilization of full postnatal care (PNC) and factors associated with full PNC in bivariate analysis.

Factors	Total, n	Full PNC, n (%)	Crude odds ratio (95% CI)	P-value
Overall	500	126 (25.20%)	21.58–29.21	
Age group of respondents				0.0004
<25 years	125	24 (19.2)	1	
25–35 years	241	80 (33.2)	2.09 (1.24–3.52)	
>35 years	134	22 (16.4)	0.83 (0.44–1.56)	
Education attainment				
Primary or below	361	58 (16.1)	1	<0.0001
Secondary or higher	139	68 (48.9)	5.00 (3.24–7.73)	
Husband’s education attainment				
Primary or below	319	46 (14.4)	1	<0.0001
Secondary or higher	181	80 (44.20)	4.70 (3.06–7.22)	
Per capita income				
<50,000 MMK	304	33 (10.9)	1	<0.0001
≥50,000 MMK	196	93 (47.5)	7.41 (4.69–11.71)	
Access to nearest health center				<0.0001
Not easy	224	36 (16.1)	1	
Easy	276	90 (32.6)	2.52 (1.63–3.91)	
Male involvement				<0.0001
No	266	19 (7.1)	1	
Yes	234	107 (45.7)	10.95 (6.43–18.66)	
Birth order				<0.0001
Second or higher	262	41 (15.7)	1	
First	238	85 (35.7)	2.99 (1.95–4.58)	
Awareness on postnatal danger signs				<0.0001
Low level	381	56 (14.7)	1	
High level	119	70 (58.8)	8.29 (5.22–13.16)	
Acceptance of TBA				<0.0001
Not accepted	256	114 (44.5)	1	
Accepted	244	12 (4.9)	0.06 (0.03–0.12)	
Type of maternal care provider				<0.0001
Non-skilled provider	227	6 (2.6)	1	
Skilled provider	273	120 (44.0)	28.9 (12.4–67.3)	
ANC visits				<0.0001
No ANC or <4 visits	238	16 (6.7)	1	
≥4 visits	262	110 (42.0)	10.04 (5.7–17.6)	
Place of delivery				<0.0001
Home	322	47 (14.6)	1	
Health center or Hospital	178	79 (44.4)	4.67 (3.04–7.16)	
Misconceptions				<0.0001
No	252	117 (46.4)	1	
Yes	248	9 (3.6)	0.04 (0.02–0.09)	

TBA, traditional birth attendant; ANC, antenatal care.

### Determinants of full PNC utilization: Multivariable analysis

After adjusting for covariates using multivariable analysis with multivariable logistic regression implemented with GEE, it was found out that the higher the degree of school education of the mother, the larger the odds of utilizing full PNC (adjusted odds ratio (AOR), 2.16; 95% CI, 1.18-3.94) ( Table 4). The rural women earning higher incomes (≥50,000 MMK) were twice as likely to receive full PNC as their counterparts earning <50,000 MMK (AOR, 2.02; 95% CI, 1.11-3.68). The participants who received support from their spouses to receive PNC were 2.19 times more likely to utilize full PNC than those who did not receive male involvement (AOR, 2.19; 95% CI, 1.02-4.69). The respondents who were knowledgeable about postnatal danger signs were two times more likely to receive full PNC than those with low awareness (AOR, 2.10; 95% CI, 1.15-3.83). Delivery of the first child (AOR, 3.26; 95% CI, 1.8-5.91) was identified as a conclusive determinant of full PNC usage. The presence of misconceptions regarding postnatal practice had a strong negative impact on the utilization of full PNC, with an AOR of 0.12 (95% CI, 0.04-0.36).

**Table 4.** Adjusted odds ratio (AOR) of factors associated with full PNC utilization with 95% CI

Factors	Total, n	Full PNC, %	Crude OR	AOR (95% CI)	P-value
Education attainment level					0.012
Primary or below	361	16.1	1	1	
Secondary or higher	139	48.9	5.0	2.16 (1.18-3.94)	
Per capita income, MMK					0.022
<50,000	304	10.86	1	1	
≥50,000	196	47.45	7.41	2.02 (1.11-3.68)	
Male involvement					0.044
No	266	7.14	1	1	
Yes	234	45.73	10.95	2.19 (1.02-4.69)	
Awareness level of postnatal danger signs					0.015
No or low	381	14.7	1	1	
High level	119	58.82	8.29	2.10 (1.15-3.83)	
Birth order					<0.0001
Second or higher	262	15.65	1	1	
First	238	35.71	2.99	3.26 (1.80-5.91)	
Misconceptions					<0.0001
No	252	46.43	1	1	
Yes	248	3.63	0.04	0.12 (0.04-0.36)	

Complete de-identified demographic information for each women taking part in the study, in addition to the answer provided to each question of the questionnaire A dictionary of terms used in the dataset is also included. Click here for additional data file. Copyright: © 2018 Mon AS et al. 2018 Data associated with the article are available under the terms of the Creative Commons Zero "No rights reserved" data waiver (CC0 1.0 Public domain dedication).

## Discussion

This community-based study was conducted to assess the extent of and determinants on full postnatal care utilization of rural Myanmar women. The present study highlighted the inadequate receipt of postnatal care among mothers in rural Myanmar. The prevalence of full PNC utilization was only 25.2%. A national survey focusing on the timing of postnatal visit revealed that the overall prevalence was 68% ^20^. The variation in presenting this utilization rate might be due to different operational definitions for outcome variable in different studies. Moreover, comparing the proportions of complete ANC attendance and health facility delivery, that of full PNC usage is markedly lower among the participants of this study. The attainment of a higher level of education was significantly associated with the receipt of full PNC in the current study, which was consistent with other studies conducted in Bangladesh and Nepal ^9, 21^ and, in addition, also homogeneous with the findings of a national survey ^20^. This might be due to the fact that mothers with higher education attainment are more likely to seek health information about safe motherhood, including newborn care, availability and accessibility to health care services from reliable sources of information. Studies undertaken in Indonesia, India and China indicated that the wealth of the mother was associated with the receipt of PNC ^11, 13, 22^. Similarly, in our study, rural mothers with low per-capita income (less than 50,000 MMK; the amount below the international poverty line as determined by the World Bank), were less likely to use full PNC. The possible explanation might that low income resulted in financial hardship, leading to barriers for taking full PNC. This explanation was strongly supported by the notion that more than two-third of non-users in this study reported they didn’t receive PNC because of unaffordability in terms of time and money.

In the present study, male involvement in spousal discussion on receipt of maternal care services and accompanying the partner to health facility was observed to have a positive influence on full PNC utilization, fitting with data from a study from India in which male involvement and their knowledge about maternal health significantly related to the maternal healthcare utilization ^23^. Regarding obstetric determinants, prior studies mentioned that factors such as birth order, knowledge about perinatal danger signs, antenatal attendance and place of delivery had association with PNC uptake ^9, 13, 14, 21, 22, 24^. This study also revealed that first birth order and high awareness of postnatal danger signs were very strong pull factors on full PNC utilization. However, unexpectedly, the frequency of antenatal attendance and place of delivery did not guarantee full PNC usage. The potential reason behind this might be the participants were not likely to be informed about the importance of PNC, its availability, recommended timing and targeted frequency of postnatal visits during antenatal visits and before discharge from health facility after delivery, leading to ignorance of PNC until mothers encountered any postnatal complication or abnormality. Moreover, in the current study, a significant proportion of rural women did not receive education and counseling relating to breastfeeding, postnatal danger signs and contraception. This indicated that there might be a weakness in delivering health messages from health care providers to rural mothers.

Consistent with prior studies on postpartum belief and practice, misconceptions regarding postnatal practice were proved as barrier to PNC uptake by the evidence from the current research ^25, 26^. Rural women who had such misconceptions exhibited 88% lower usage of full PNC than those who did not. Based on Myanmar customs and traditional beliefs, food prohibition, behavioral restriction or both within the postpartum period were observed among nearly half of the participants (49.5%). Breastfeeding mothers who had postpartum food taboos perceived that meat consumption could make the newborn ill and that some vegetables, such as roselles, cause abdominal pain and flatulence for both mother and baby. Some mothers reported that they ate only fried fish, dried fish, dried prawns and soup during their postpartum period. This food avoidance practice might result in nutritional deficiency for both mothers and babies. Another common misconception perpetuated among rural women was that strict home confinement within 7 days after delivery; this behavioral restriction might bar to timely and adequate attendance of PNC.

This study has a number of strengths. This is the first study to reveal the prevalence and determinants of utilization of full PNC, based on the recommended timing and frequency of postnatal visits as per updated WHO postnatal guideline, among rural Myanmar women. Our data analysis, developed using our aforementioned sampling technique, is thereby more likely to provide valid estimates. In addition, the evidence obtained from the current research provides updated knowledge and assistance for the policy makers and healthcare providers to extend quality maternal healthcare package nationwide. Nonetheless, the present study has some limitations. The cross-sectional nature restricts the ability to draw cause-effect relationships between the potential predictors and full PNC utilization. Since the participants were reporting past experience and practice, there may have elicited recall bias. Nevertheless, a 2-year recall period was selected to minimize this bias.

## Conclusion

The current study reported on the underutilization of postnatal care among rural Myanmar women. The key determinants on full PNC were education attainment, having higher income, male involvement, the first birth order, awareness of postnatal danger signs, and presence of postnatal misconception. On the basis of the evidence generated in this study, coverage of maternal healthcare emphasizing PNC should be intensified to reach out to less-educated mothers, those from low-income families and high-birth-order mothers. An awareness-raising program highlighting the importance and availability of postnatal care is essential to improve full PNC utilization; it is urgently needed to facilitate the health care providers for provision of essential and updated health information concerning safe motherhood and newborn care, in order to correct harmful misconceptions and upgrade knowledge regarding perinatal danger signs among rural women. Further study focusing on quality of PNC services and satisfaction on services the rural women received should be recommended.

## Data availability

The data referenced by this article are under copyright with the following copyright statement: Copyright: © 2018 Mon AS et al.

Data associated with the article are available under the terms of the Creative Commons Zero "No rights reserved" data waiver (CC0 1.0 Public domain dedication). 

**Dataset 1. Complete de-identified demographic information for each women taking part in the study, in addition to the answer provided to each question of the questionnaire.** A dictionary of terms used in the dataset is also included. DOI: https://doi.org/10.5256/f1000research.15561.d211750 ^19^.

## Supplementary material

Supplementary File 1. The questionnaire given to each woman in this study, in the original Burmese and English.
