# Supplementary material for: Utilization of full postnatal care services among rural Myanmar women and its determinants: a cross-sectional study
Source: F1000Res. 2018 Aug 1;7:1167. [Version 1] doi: 10.12688/f1000research.15561.1 (PMC6085599; doi:10.12688/f1000research.15561.1)
Supplement: Complete de-identified demographic information for each women taking part in the study, in addition to the answer provided to each question of the questionnaire — A dictionary of terms used in the dataset is also included. [file f1000research-7-16974-s0000.tgz › eec75aad-107c-4158-90c8-35050c3809c7_Dataset.zip!/Dataset/4.Data dictionary.docx]

**Operational definition of variables and data dictionary**

| **No.** | **Variables’ names** | **Description of variables** | **Code** |
| --- | --- | --- | --- |
| 1 | id | identification number of respondents | numerical value |
| 2 | vil | villages | numerical value |
| 3 | age | age of respondents | numerical value |
| 4 | agegp | age group of respondents | 1= < 25 years  2= 25 to 35 years  3= more than 35 years |
| 5 | edu | education attainment of respondents | 1= illiterate  2= can read & write  3= primary school  4= middle school  5= high school  6= university  7= graduated & higher |
| 6 | edugp | recode of “edu” for analysis and include 2 levels  1) primary or below level; composed of ‘illiterate’ ‘can read & write’ and ‘primary’  2) secondary or above level; composed of  ‘secondary’ ‘high’ ‘university’ and ‘graduates & above’ | 0= primary or below  1= secondary or above |
| 7 | edu_hus | education attainment of their husbands | 1= illiterate  2= can read & write  3= primary school  4= middle school  5= high school  6= university  7= graduated & higher |
| 8 | edugp_hus | recode of “edu_hus” for analysis and include 2 levels and identical groups to “edugp” | 0= primary or below  1= secondary or above |
| 9 | order | birth order i.e. the order of a child is born to his/her family (discrete numerical nature) | numerical value |
| 10 | order_1 | birth order group; recode of “order” | 0= second or higher birth order  1= first birth order |
| 11 | income | average monthly family income | numerical value |
| 12 | family | number of family members | numerical value |
| 13 | percapita | average monthly per-capita income | numerical value |
| 14 | percapita_gp | average monthly per-capita income group | 0= < 50,000 Myanmar kyats (MMK)  1= >= 50,000 MMK |
| 15 | access | accessibility to nearest health center | 0= not easy  1= easy in access |
| 16 | minvolve | male involvement | 0= absent  1= present |
| 17 | pntotal | total knowledge scores regarding 8 postpartum danger signs (1 mark for recognizing 1 sign) | numerical value |
| 18 | neototal | total knowledge scores regarding 6 neonatal danger signs (1 mark for recognizing 1 sign) | numerical value |
| 19 | awareness | awareness on postnatal danger signs and include 2 levels  ‘high level’= could mention at least 3 postpartum danger signs and at least 1 neonatal danger sign.  otherwise, termed as ‘low level’ | 0= low level  1= high level |
| 20 | accept_tba | acceptance of TBA as a skilled attendant | 0= not accepted  1= accepted |
| 21 | provider | types of healthcare provider | 0= non-skilled provider  1= skilled provider |
| 22 | anctimes | numbers of antenatal (AN) visits | numerical value |
| 23 | ancvisit | group of numbers of AN visits | 0= no ANC or < 4 AN visits  1= >= 4 AN visits |
| 24 | birthplace | place of delivery | 0= home  1= hospital/ health center |
| 25 | pn | whether the women had taken PNC or not | 0= no  1= yes |
| 26 | pntimes | numbers of PN visits | numerical value |
| 27 | pnfirst | timing of first PN visit | 1= within 24 hours  2= 24 to 48 hours  3= 48 to 72 hours  4= 3 to 7 days  5= after 7 days |
| 28 | wound | receipt of maternal check up (wound care) | 0= not received  1= received |
| 29 | bf | receipt of counseling on breastfeeding | 0= not received  1= received |
| 30 | contrahe | receipt of counseling on contraceptives | 0= not received  1= received |
| 31 | contra | receipt of contraceptives provision | 0= not received  1= received |
| 32 | neocare | receipt of neonatal care | 0= not received  1= received |
| 33 | pnhe | receipt of health education about postnatal danger signs | 0= not received  1= received |
| 34 | b1 | receipt of postnatal nutritional supplements provision | 0= not received  1= received |
| 35 | pncompli | postnatal complications | 0= no  1= yes |
| 36 | pnfood | Food avoidance within postnatal period | 0= no  1= yes |
| 37 | pnbehave | Behavioural restriction within postnatal period | 0= no  1= yes |
| 38 | pnmiscon | Misconceptions on postnatal practices | 0= no  1= yes |
| 39 | fullpnc | utilization of full PNC | 0= not received  1= received |
