# Supplementary material for: Utilization of full postnatal care services among rural Myanmar women and its determinants: a cross-sectional study
Source: F1000Res. 2018 Aug 1;7:1167. [Version 1] doi: 10.12688/f1000research.15561.1 (PMC6085599; doi:10.12688/f1000research.15561.1)
Supplement: Supplementary file 2 [file f1000research-7-16974-s0001.tgz › e89f9c09-8fd3-48c5-9e89-9514ceda1ce7.zip!/Supplement/2_2.Questionnaires_English.pdf]

Questionnaire for Utilization of Maternal Healthcare Services among Rural Myanmar Women; Determinants and Rates

Date of interview: ------/------/-----

	Question	Coding Categories	Code	Skip
**Section 1: Socio-demographic Characteristics**				
1	Identification number	ID---------------------------		
2	Name of township	--------------------------------		
3	Name of village	--------------------------------		
4	How old are you?	Completed age in years---------------		
5	What is your race?	Bamar<br>Others (specify)------------	1<br>2	
6	What is your religion?	Buddhist<br>Others(specify)-----------	1<br>2	
7	What is your highest level of education?	Illiterate<br>Read and Write<br>Primary<br>Middle<br>High<br>University / college / institute<br>Graduate / Post. Graduate	1<br>2<br>3<br>4<br>5<br>6<br>7	
8	What is your husband's education?	Illiterate<br>Read and Write<br>Primary<br>Middle<br>High<br>University / college / institute<br>Graduate / Post. Graduate	1<br>2<br>3<br>4<br>5<br>6<br>7	
9	How many persons in your family?	Family members--------------------		
10	How much average monthly family income?	Income (kyats/ month)----------------		
11	Who lives with you in your home?<br>(Multiple response)	Father<br>Mother<br>Father-in-law<br>Mother-in-law<br>Husband<br>Wife (yourself)<br>Relatives<br>Children<br>Others (specify) --------------	1<br>2<br>3<br>4<br>5<br>6<br>7<br>8<br>9	

12	Did you discuss with your husband in taking maternal services eg, contraception, care during perinatal period?	Yes<br>No	1<br>2	
13	Did your husband accompany you for taking maternal care?	Yes<br>No	1<br>2	
**Section 2: Access to skilled maternal care**				
14	How long does it take to reach the nearest health facility?	Within 30 minutes<br>30 mins to 1 hr<br>1 hr to 1 hr and 30 mins<br>More than 1 hr and 30 mins	1<br>2<br>3<br>4	
15	Which mode of transport do you use to go to the nearest health facility?	Walking<br>Cart<br>Motorcycle<br>Car	1<br>2<br>3<br>4	
16	Can you go there without difficulty at any times?	Yes<br>No	1<br>2	
17	Can you afford the cost of transportation to there?	Yes<br>No	1<br>2	
18	Can you afford the cost of maternal healthcare services?	Yes<br>No	1<br>2	
**Section 3: Knowledge on danger signs of each perinatal period and perception on TBA**				
19	What are the danger signs during pregnancy?<br>(not prompted, multiple responses allowed)	Severe headache<br>High fever<br>Fits/Unconsciousness<br>Severe anaemia/Pallor<br>Swelling of face and limbs<br>Difficulty in breathing<br>Blurred vision<br>Dribbling<br>Bleeding per vagina<br>Don’t know<br>Others (specify)--------------------	1<br>2<br>3<br>4<br>5<br>6<br>7<br>8<br>9<br>10<br>11	

20	What are the danger signs during childbirth?<br>(not prompted, multiple responses allowed)	Severe headache<br>High fever<br>Fits/Unconsciousness<br>Excessive bleeding<br>Prolonged labour<br>Difficulty in breathing<br>Retained placenta<br>Pre-mature rupture of membrane<br>Don’t know<br>Others (specify)------------------	1<br>2<br>3<br>4<br>5<br>6<br>7<br>8<br>9<br>10	
21	What are the danger signs during postpartum?<br>(not prompted, multiple responses allowed)	High fever<br>Severe headache<br>Blurred vision<br>Difficulty in breathing<br>Fits/Unconsciousness<br>Postpartum haemorrhage<br>Foul smelling vaginal discharge<br>Breast engorgement/ Mastitis<br>Don’t know<br>Others (specify)------------------------	1<br>2<br>3<br>4<br>5<br>6<br>7<br>8<br>9<br>10	
22	What are the danger signs of newborn?	Cannot suck well<br>Neonatal jaundice<br>Fever<br>Fits<br>Reddish coloration of umbilical area<br>Umbilical sepsis<br>Don’t know<br>Others (specify)-----------------	1<br>2<br>3<br>4<br>5<br>6<br>7<br>8	
23	Who are the skilled providers needed for perinatal care?	Doctor<br>Nurse<br>LHV<br>Midwife<br>AMW<br>TBA<br>Don’t know<br>Others (specify)---------------	1<br>2<br>3<br>4<br>5<br>6<br>7<br>8	
24	Do you think it is safe for taking maternal care by TBA?	Yes<br>No<br>Don’t know	1<br>2<br>3	
25	If response ‘Yes’, why?	----------------------------------		

History and Practices during preinatal period of the most recent child				
**Section 4: Antenatal Care History and Practice including birth preparedness**				
26	What was your age at the most recent child pregnancy?	Age in years---------------		
27	What was the birth order of the most recent child?	--------------------------------		
28	How old is the most recent child?	Age in months-----------------------		
29	Did you receive ANC from the skilled provider during the recent pregnancy?	Yes<br>No	1<br>2→	 39
30	Who was your skilled care provider?	Doctor<br>Nurse<br>LHV<br>Midwife<br>Others (specify)-----------------	1<br>2<br>3<br>4<br>5	
31	Where did you take ANC?	Public hospital<br>Private hospital/ clinic<br>Rural health center<br>Sub center<br>Others (specify)--------------	1<br>2<br>3<br>4<br>5	
32	When did you start taking first antenatal visit?	Weeks in pregnancy ----------------		
33	How many visits did you take?	--------------------------------		
34	Which health services did you get during your AN visits?	Physical examination (including weight, blood pressure, heart rate)<br>Gynaecological examination<br>Blood/ Urine tests<br>PMCT counselling<br>Nutritional supplements<br>Tetanus vaccine<br>Health Education	1<br>2<br>3<br>4<br>5<br>6<br>7	
35	Did you take iron and folic acid tablets during last pregnancy?	Yes<br>No	1<br>2→	 37
36	(If yes) how have you taken?	Daily<br>Others (specify)--------------	1<br>2	
37	Did you receive TT injection?	Yes<br>No	1<br>2→	 41
38	(If yes) how many times?	--------------------------------		

39	Why did not take ANC from skilled provider?	Not afford for cost<br>Transportation difficulty<br>Dissatisfaction of the staff's service<br>Assurance of normal pregnancy<br>Advice from husband/ relatives<br>Acceptance on TBA<br>Others (specify)-------------	1<br>2<br>3<br>4<br>5<br>6<br>7	
40	Who gave ANC if you did not take ANC from skilled provider?	AMW<br>TBA<br>None<br>Others (specify)----------------	1<br>2<br>3<br>4	
41	Did you experience any complication during recent pregnancy?	Yes<br>No	1<br>2	
42	If yes, please mention the complication.	------------------------------------		
43	Did you perform food prohibition or any special behavioural constraint during pregnancy?	Food prohibition<br>(specify)---------------------------------<br>Behavioural constraint<br>(specify)---------------------------------<br>None of the above	1<br><br>2<br><br>3	
**Section 5: Intranatal Care History and Practices**				
44	Where did you deliver your most recent child?	Public hospital<br>Private hospital/ clinic<br>Rural health center<br>Sub center<br>Home<br>Others (specify)-----------------	1<br>2<br>3<br>4<br>5<br>6	
45	Who decided the delivery place?	Self<br>Husband<br>Female relatives superior<br>Others (specify)-------------------	1<br>2<br>3<br>4	
46	Who assisted your recent delivery?	Doctor<br>Nurse<br>LHV<br>Midwife<br>AMW<br>TBA<br>Others (specify)-----------------	1<br>2<br>3<br>4<br>5<br>6<br>7	

47	What was the mode of delivery for the recent child?	Normal delivery<br>Forceps/ Vacuum<br>LSCS	1<br>2<br>3	
48	Was there any complication during childbirth?	Yes<br>No	1<br>2	
49	If yes, please mention the complication.	-------------------------------		
**Section 6: Postnatal Care History and Practices**				
50	Did you receive postnatal care?	Yes<br>No	1<br>2 →	 54
51	Where did you take postnatal care?	Public hospital<br>Private hospital/ clinic<br>Rural health center<br>Sub center<br>Home<br>Others (specify)-----------------	1<br>2<br>3<br>4<br>5<br>6	
52	When did you start getting care?	Within 24 hours<br>24 to 48 hours<br>48 to 72 hours<br>After 72 hours<br>After 7 days	1<br>2<br>3<br>4<br>5	
53	How many visits did you take?	-------------------------		
54	Who was your postnatal care provider?	Doctor<br>Nurse<br>HA<br>LHV/ midwife<br>AMW<br>TBA<br>Others (specify)-----------------	1<br>2<br>3<br>4<br>5<br>6<br>7	
55	Which services did you receive during your postnatal visits?	Physical examination<br>Counseling on breastfeeding<br>Counseling on contraceptives<br>Providing contraceptive<br>Neonatal care<br>HE on danger signs of postpartum<br>Nutritional supplements	1<br>2<br>3<br>4<br>5<br>6<br>7	
56	Did you experience any complications after delivery?	Yes<br>No	1<br>2	

57	If yes, please mention the complication.	PPH<br>Fever<br>Infection of the wound<br>Mastitis/ Breast abscess<br>Others (specify)-----------------	1<br>2<br>3<br>4<br>5	
58	Did you perform food prohibition or any traditional related behavioural constraint during postpartum?	Food prohibition<br>(specify)--------------------------------<br>Behavioural constraint<br>(specify)---------------------------------<br>None of the above	1<br><br>2<br><br>3	
