# Supplementary material for: Utilization of full postnatal care services among rural Myanmar women and its determinants: a cross-sectional study
Source: F1000Res. 2018 Aug 1;7:1167. [Version 1] doi: 10.12688/f1000research.15561.1 (PMC6085599; doi:10.12688/f1000research.15561.1)
Supplement: Supplementary file 2 [file f1000research-7-16974-s0001.tgz › e89f9c09-8fd3-48c5-9e89-9514ceda1ce7.zip!/Supplement/3_2.Questionnaires_Myanmar.pdf]

ကျေးလက်နေ မြန်မာအမျိုးသမီးများ၏ မိခင်ဘ၀ကျန်းမာရေးစောင့်ရှောက်မှုခံယူခြင်း အပေါ်တွင်လွှမ်းမိုးသည့်အချက်အလက်များနှင့် စောင့်ရှောက်မှုခံယူသူနှုန်းနှင့် ပတ်သက်သောအချက်အလက်ဆိုင်ရာ သုတေသနမေးခွန်းလွှာ

အပိုင်း(၁) ဖြေဆိုသူ၏ နောက်ခံအကြောင်းအရာ

၁။ အမှတ်စဉ် ---------------------------

၂။ ကျေးရွာအမည် ---------------------------

၃။ မြို့ ---------------------------

၄။ ပြည့်ပြီးအသက်(နှစ်) --------------------------

၅။ လူမျိုး --------------------------

(၁) ဗမာ (၂) အခြား (ဖော်ပြရန်) --------------------------

၆။ ကိုးကွယ်သည့်ဘာသာ

(၁) ဗုဒ္ဓ (၂) အခြား(ဖော်ပြရန်) --------------------------

၇။ ဖြေဆိုသူ၏ အမြင့်ဆုံး ပညာအရည်အချင်း

(၁) စာမတတ်/ကျောင်းမနေဘူး

(၂) ရေးတတ်/ဖတ်တတ်/ဘုန်းကြီးကျောင်း

(၃) မူလတန်း (၄) အလယ်တန်း

(၅) အထက်တန်း (၆) တက္ကသိုလ်

(၇) ဘွဲ့ရ/ဘွဲ့လွန်

၈။ ခင်ပွန်း၏ အမြင့်ဆုံးပညာအရည်အချင်း

(၁) စာမတတ်/ကျောင်းမနေဘူး

(၂) ရေးတတ်/ဖတ်တတ်/ဘုန်းကြီးကျောင်း

(၃) မူလတန်း (၄) အလယ်တန်း

(၅) အထက်တန်း (၆) တက္ကသိုလ်

(၇) ဘွဲ့ရ/ဘွဲ့လွန်

၉။ မိသားစုဝင်ဦးရေ ----------------------------------- ဦး

၁၀။ မိသားစု၏ လစဉ်ပျမ်းမျှဝင်ငွေ ------------------------------------ ကျပ်

၁၁။ သင်၏အိမ်တွင် ဘယ်သူတွေ အတူနေထိုင်ပါသလဲ။
(တစ်ခုမက ဖိုင်းနိုင်သည်)

(၁) ဖခင် (၂) မိခင် (၃) ယောက္ခထီး

(၄) ယောက္ခမ (၅) ခင်ပွန်း (၆) ဇနီး (ဖြေဆိုသူ)

(၇) သားသမီး (၈) ဆွေမျိုးများ (၉) အခြား (ဖော်ပြရန်) --------------------------

၁၂။ မိခင်ကျန်းမာရေး စောင့်ရှောက်မှု ခံယူခြင်းနှင့် ပတ်သက်၍ သင်၏ခင်ပွန်းနှင့် တိုင်ပင် ဆွေးနွေးခြင်းရှိပါသလား။

(၁) ရှိပါသည်။ (၂) မရှိပါ

၁၃။ မိခင်ကျန်းမာရေးစောင့်ရှောက်မှု ခံယူရန်အတွက် ကျန်းမာရေးဌာနသို့ သွားလျှင် သင်၏ခင်ပွန်းက သင့်ကို လိုက်ပါပို့ဆောင်ခြင်းရှိပါသလား။

(၁) ရှိပါသည်။ (၂) မရှိပါ

အပိုင်း(၂) မိခင်ကျန်းမာရေး စောင့်ရှောက်မှု လက်လှမ်းမီခြင်းဆိုင်ရာ မေးခွန်းများ

၁၄။ အနီးဆုံးကျန်းမာရေးဌာနသို့ သွားရောက်ရန် ဘယ်လောက်ကြာပါသလဲ။

(၁) နာရီဝက်အတွင်း (၂) နာရီဝက်မှတစ်နာရီကြာ

(၃) တစ်နာရီမှ တစ်နာရီခွဲကြာ (၄) တစ်နာရီခွဲအထက်

၁၅။ အနီးဆုံးကျန်းမာရေးဌာနသို့ ဘယ်လိုသွားလေ့ရှိပါသလဲ။

(၁) ခြေချင် (၂) လှည်း (မြင်းလှည်း၊နွားလှည်း)

(၃) ဆိုင်ကယ်/ထော်လာဂျီ (၄) မော်တော်ကား

၁၆။ အချိန် ၊ ရာသီမရွေး အခက်အခဲမရှိသွားလာနိုင်ပါသလား။

(၁) သွားနိုင်သည် (၂) မသွားနိုင်ပါ

၁၇။ အနီးဆုံး ကျန်းမာရေးဌာန သို့သွားရောက်ရန်ခရီး စရိတ်တတ်နိုင်ပါသလား။

(၁) တတ်နိုင်သည်။ (၂) မတတ်နိုင်ပါ။

၁၈။ အနီးဆုံး ကျန်းမာရေးဌာနတွင် မိခင်ဘဝကျန်းမာရေး စောင့်ရှောက်မှုခံယူရန် စရိတ်တတ်နိုင်ပါသလား။

(၁) တက်နိုင်သည်။ (၂) မတတ်နိုင်ပါ။

အပိုင်း(၃) ကိုယ်ဝန်ဆောင်ချိန်၊မီးဖွားချိန်နှင့် မီးဖွားပြီးချိန်တို့တွင် တွေ့ကြုံတတ်သည့် အန္တရာယ်လက္ခဏာများနှင့် ပတ်သက်သော ဗဟုသုတဆိုင်ရာ မေးခွန်းများ

(မေးခွန်း (၁၉) မှ (၂၃) အားတစ်ခုထက်ပို၍ ဖြေဆိုနိုင်သည်။အဖြေမဖတ်ပြရပါ။)

၁၉။ ကိုယ်ဝန်ဆောင်ချိန်တွင် သတိပြုရမည့်အန္တ ရာယ်လက္ခဏာတွေကဘာတွေလဲ။

(၁) ပြင်းထန်စွာခေါင်းကိုက်ခြင်း (၂) ပြင်းထန်စွာဖျားခြင်း

(၃) တက်ခြင်း/သတိလစ်ခြင်း (၄) အလွန်သွေးအားနည်းခြင်း/ဖြူဖျော့ခြင်း

(၅) အသက်ရှူမြန်ခြင်း/ အသက်ရှူခက်ခဲခြင်း (၆) ခြေ၊လက်၊မျက်နှာ ဖောယောင်ခြင်း

(၇) အမြင်မှုန်ဝါးခြင်း (၈) မိန်မကိုယ်မှ ရေဆင်းခြင်း

(၉) မိန်းမကိုယ်မှ သွေးဆင်းခြင်း (၁၀) မသိပါ။

(၁၁) အခြား(ဖော်ပြရန်) -------------------------

၂၀။ မွေးဖွားချိန်တွင်သတိပြုရမည့် အန္တ ရာယ်လက္ခဏာတွေကဘာတွေလဲ။

(၁) ပြင်းထန်စွာခေါင်းကိုက်ခြင်း (၂) ပြင်းထန်စွာဖျားခြင်း

(၃) တက်ခြင်း/သတိလစ်ခြင်း (၄) မွေးဖွားစဉ် သွေးဆင်းများခြင်း

(၅) ဗိုက်စနာချိန်မှစ၍ (၁၂)နာရီကြာသည့်တိုင်အောင် ကလေးမမွေးနိုင်ခြင်း

(၆) အသက်ရှူမြန်ခြင်း/ အသက်ရှူခက်ခဲခြင်း

(၇) မွေးဖွားပြီး (၁)နာရီအတွင်း အချင်းမကျခြင်း

(၈) ရေမွှာပေါက်ပြီး (၆)နာရီကြာသည့်တိုင်အောင် ဗိုက်မနာခြင်း

(၉) မသိပါ (၁၀) အခြား(ဖော်ပြရန်) -------------------------

၂၁။ မွေးဖွားပြီးချိန်တွင်သတိပြုရမည့်အန္တရာယ်လက္ခဏာတွေကဘာတွေလဲ။

(၁) ပြင်းထန်စွာခေါင်းကိုက်ခြင်း (၂) ပြင်းထန်စွာဖျားခြင်း

(၃) အမြင်မှုန်ဝါးခြင်း (၄) တက်ခြင်း/သတိလစ်ခြင်း

(၅)အသက်ရှူမြန်ခြင်း/ အသက်ရှူခက်ခဲခြင်း (၆) မွေးဖွားပြီးနောက် သွေးသွန်ခြင်း

(၇) မိန်းမကိုယ်မှ ပုပ်နံသော အရည်ဆင်းခြင်း

(၈) သားမြတ် (သို့) နို့သီးခေါင်းရောင်ခြင်း

(၉) မသိပါ

(၁၀) အခြား(ဖော်ပြရန်) -------------------------

၂၂။ မွေးကင်းစကလေးငယ်တွေရဲ့ စိုးရိမ်ရသော လက္ခဏာတွေက ဘာတွေလဲ။

(၁) ကလေးနို့မစို့နိုင်ခြင်း (၂) ကလေးအသားဝါခြင်း

(၃) ဖျားခြင်း (၄) ကလေးတက်ခြင်း

(၅) ချက်ပတ်လည်အရေပြားနီရဲခြင်း (၆) ချက်ပြည်တည်ခြင်း

(၇) မသိပါ (၈) အခြား(ဖော်ပြရန်) --------------------------

၂၃။ ကိုယ်ဝန်ဆောင်ချိန်၊မီးဖွားချိန်နှင့် မီးဖွားပြီးချိန်တွင် ကျွမ်းကျင်ကျန်းမာရေးစောင့်ရှောက်မှုပေးသူဆိုတာ ဘယ်သူတွေလဲ။

(၁) ဆရာဝန် (၂) သူနာပြုဆရာမ

(၃) အမျိုးသမီးကျန်းမာရေးဆရာမ (၄) သားဖွားဆရာမ

(၅) အရန်သားဖွား (၆) လက်သည်

(၇) မသိပါ (၈) အခြား (ဖော်ပြရန်)------------------------------

၂၄။ လက်သည်နှင့် ကိုယ်ဝန်အပ်ခြင်း မွေးဖွားခြင်းတို့ပြုလုပ်ခြင်းသည် အန္တရာယ်ကင်း စိတ်ချရသည်ဟုထင်ပါသလား။

(၁) ထင်ပါသည် (၂) မထင်ပါ (၃) မဖြေတတ်ပါ

၂၅။ အကယ်၍ ထင်ပါသည်ဟု ဖြေဆိုပါက ဘာကြောင့်စိတ်ချရသည်ဟု ထင်ပါသလဲ။

အငယ်ဆုံးကလေး(သို့) နောက်ဆုံးကလေး၏ မွေးဖွားခြင်းရာဇဝင်

အပိုင်း(၄) ကိုယ်ဝန်ဆောင် စောင့်ရှောက်မှုခံယူခြင်းနှင့် မမွေးဖွားမီကြိုတင်ပြင်ဆင်ခြင်း

၂၆။ အငယ်ဆုံးကလေး(သို့) ယခုကလေးကိုယ်ဝန်ဆောင်စဉ်မှာ အသက်ဘယ်လောက်ရှိပြီလဲ။ --နှစ်

၂၇။ ဒီကလေးက ဘယ်နှစ်ယောက်မြောက်ကလေး ဖြစ်ပါသလဲ။ -------------------------

၂၈။ ဒီကလေးက အသက်ဘယ်လောက်ရှိသွားပြီလဲ။ ----------------------------------လ

၂၉။ ဒီကလေး ကိုယ်ဝန်ဆောင်ချိန်မှာ ကျွမ်းကျင်ကျန်းမာရေးဝန်ထမ်းတွေနဲ့ ကိုယ်ဝန်အပ်ခြင်းပြုလုပ်ခဲ့ပါသလား။

(၁) ပြုလုပ်ခဲ့ပါသည် (၂) မပြုလုပ်ခဲ့ပါ

မပြုလုပ်ခဲ့ပါဟု ဖြေဆိုလျှင် မေခွန်း (၃၉)သို့ကျော်၍ မေးရန်

၃၀။ ကိုယ်ဝန်ဆောင်စောင့်ရှောက်မှုပေးသော ကျန်းမာရေးဝန်ထမ်းမှာ ဘယ်သူပါလဲ။

(၁) ဆရာဝန် (၂) သူနာပြုဆရာမ

(၃) အမျိုးသမီးကျန်းမာရေးဆရာမ (၄) သားဖွားဆရာမ

(၅) အခြား(ဖော်ပြရန်) --

၃၁။ ကိုယ်ဝန်အပ်ခြင်းကို မည်သည့်နေရာတွင် ပြုလုပ်ခဲ့ပါသလဲ။

(၁) အစိုးရဆေးရုံ (၂) ပုဂ္ဂလိကဆေးရုံ/ဆေးခန်း

(၃) ကျေးလက်ကျန်းမာရေးဌာန (RHC) (၄) ကျေးလက်ကျန်းမာရေးဌာနခွဲ(SC)

(၅) အခြား(ဖော်ပြရန်) --

၃၂။ ကိုယ်ဝန်သက်တမ်း ဘယ်လောက်ကြာမှာ စပြီးကိုယ်ဝန်အပ်ခဲ့ပါသလဲ။

-- လ

၃၃။ ကိုယ်ဝန်စောင့်ရှောက်မှု ရရှိခဲ့သော အကြိမ်အရေအတွက် ------------------------------------

၃၄။ ကိုယ်ဝန်အပ်တဲ့ အချိန်မှာရရှိတဲ့ ကျန်းမာရေး စောင့်ရှောက်မှုများ(လိုအပ်လျှင်ဖတ်ပြနိုင်သည်)

(၁) သွေးပေါင်ချိန်ခြင်း၊ အရပ်တိုင်းခြင်း၊ ကိုယ်အလေးချိန်ချိန်ခြင်း

(၂) ဗိုက်စမ်းခြင်း၊ ကလေးနှလုံးခုန်သံနားထောင်ခြင်း

(၃) သွေးစစ်ခြင်း ၊ ဆီးစစ်ခြင်း

(၄) မိခင်မှကလေးသို့ HIV ပိုးရှိမရှိ စစ်ဆေးခြင်း

(၅) သံဓါတ်ပါသောအားဆေးများပေးခြင်း

(၆) မေးခိုင်ကာကွယ်ဆေးထိုးခြင်း

(၇) ကျန်းမာရေး အသိပညာပေး ဟောပြောခြင်း

၃၅။ ကိုယ်ဝန်ဆောင်စဉ်မှာ သံဓါတ်အားဆေးနှင့် ဖောလစ်အက်ဆစ် အားဆေးတို့ကို သောက်ခဲ့ပါသလား။

(၁) သောက်ခဲ့သည်။ (၂) မသောက်ပါ။

၃၆။ သောက်လျှင် ဘယ်လို သောက်ပါသလဲ။

(၁) နေ့စဉ် (၂) အခြား(ဖော်ပြရန်) ----------------------------

၃၇။ မေးခိုင်ကာကွယ်ဆေး ထိုးခဲ့ပါသလား

(၁) ထိုးသည် (၂) မထိုးပါ။

၃၈။ ထိုးခဲ့လျှင် မေးခိုင်ကာကွယ်ဆေး အထိုးခံခဲ့သည့် အကြိမ် -----------------------------

၃၉။ ကျွမ်းကျင်ကျန်းမာရေး ဝန်ထမ်းတွေနဲ့ဗိုက်မအပ်ခဲ့လျှင် ဘာကြောင့် မအပ်ခဲ့ပါသလဲ၊

(၁) ငွေကြေးမတတ်နိုင်လို့ (၂) သွားလာရ အဆင်မပြေလို့

(၃) ဆေးရုံ/ဆေးခန်းကိုကြောက်လို့ (၄) ပုံမှန်ကိုယ်ဝန်ဟု ယူဆလို့

(၅) ခင်ပွန်း(သို့) ဆွေမျိုးများမှမအပ်ရန်ပြောလို့ (၆) လက်သည်ကိုယုံကြည်လို့

(၇) အခြား (ဖော်ပြရန်) ---

၄၀။ ကျွမ်းကျင်ကျန်းမာရေးဝန်ထမ်းနဲ့ ဗိုက်မအပ်လျှင် အခြားဘယ်သူနဲ့ရော ဗိုက်အပ်ထားတာမျိုး ရှိသလဲ။

(၁) အရန်သားဖွားဆရာမ (၂)အရပ်လက်သည်

(၃) မရှိပါ။ (၄)အခြားဖော်ပြရန်။

၄၁။ အငယ်ဆုံးကလေးကိုယ်ဝန်ဆောင်စဉ်မှာ ကိုယ်ဝန်နဲ့ဆိုင်တဲ့ ပြဿနာ(သို့) အန္တရာယ် ကြုံတွေ့တာမျိုး ရှိပါသလား။

(၁) ရှိပါသည်။ (၂) မရှိပါ။

၄၂။ ရှိပါသည် ဟုဖြေပါက ဘယ်လိုပြဿနာမျိုးကြုံတွေ့ခဲ့တယ်။

၄၃။ ကိုယ်ဝန်ဆောင်စဉ်မှာ အစားအသောက်ရှောင်ခြင်းမျိုးနှင့် (သို့) အနေအထိုင်၊ အပြုအမူ ကန့်သတ်ဆင်ခြင်ခြင်း မျိုး ရှိပါသလား။

(၁)အစားအသောက်ရှောင်ခြင်းရှိသည်။ (ဖော်ပြရန်) --

(၂) အနေအထိုင်၊ အပြုအမူကန့်သတ်ဆင်ခြင်ခြင်းရှိသည်။ (ဖော်ပြရန်) -----------------------

(၃) နှစ်မျိုးစလုံးမရှိပါ။

အပိုင်း (၅)ကလေးမီးဖွားချိန်ကျန်းမာရေး စောင့်ရှောက်မှုခံယူခြင်း ဆိုင်ရာ မေးခွန်းများ

၄၄။ အငယ်ဆုံးကလေး (သို့)ယခုကလေးကို ဘယ်မှာ မွေးခဲ့ပါသလဲ။

(၁) အစိုးရဆေးရုံ (၂)ပုဂ္ဂလိက ဆေးရုံ/ဆေးခန်း

(၃) ကျေးလက်ကျန်းမာရေးဌာန (၄) ကျေးလက်ကျန်းမာရေးဌာနခွဲ

(၅) မိမိနေအိမ် (၆) အခြား (ဖော်ပြရန်) ----------------

၄၅။ အဲဒီနေရာမှာ မွေးဖို့ ဘယ်သူကဆုံးဖြတ်တာလဲ။

(၁)ဖြေဆိုသူကိုယ်တိုင် (၂) ခင်ပွန်း

(၃) မိဘ/ဆွေမျိုးများ/ ယောက္ခမ (၄) အခြား (ဖော်ပြရန်) -----------------------

၄၆။ အငယ်ဆုံးကလေးကို ဘယ်သူမွေးပေးခဲ့လဲ။

(၁) ဆရာဝန် (၂) သူနာပြုဆရာမ

(၃) အမျိုးသမီးကျန်းမာရေးဆရာမ (၄) သားဖွားဆရာမ

(၅) အရန်သားဖွား (၆) လက်သည်

(၇) အခြား (ဖော်ပြရန်) --

၄၇။ အငယ်ဆုံးကလေး ကို ဘယ်လိုမွေးခဲ့ပါသလဲ။

(၁) ရိုးရိုးမွေး (၂) ညှပ်ဆွဲ /လေစုပ်မွေး (၃) ဗိုက်ခွဲမွေး

၄၈။ ကလေးမွေးဖွားစဉ် အခက်အခဲ (သို့) နောက်ဆက်တွဲပြဿနာ များရှိခဲ့ပါသလား။

(၁) ရှိခဲ့သည်။ (၂)မရှိပါ။

၄၉။ ရှိခဲ့လျှင် (ဖော်ပြရန်) --

အပိုင်း (၆) မီးဖွားပြီးချိန် မီးတွင်းကာလစောင့်ရှောက်မှုခံယူခြင်း နှင့် ဆိုင်သည့် မေးခွန်းများ

၅၀။ အငယ်ဆုံးကလေးမီးဖွားပြီးချိန် မီးတွင်းကာလကျန်းမာရေး စောင့်ရှောက်မှုခံယူခြင်း ရှိပါသလား။

(၁) ရှိသည်။ (၂)မရှိပါ။

၅၁။ မီးဖွားပြီးချိန် ကျန်းမာရေးစောင့်ရှောက်မှုကို ဘယ်မှာယူခဲ့သလဲ။

(၁) အစိုးရဆေးရုံ (၂) ပုဂလိကဆေးရုံ/ ဆေးခန်း

(၃) ကျေးလက်ကျန်းမာရေးဌာန (၄) ကျေးလက်ကျန်းမာရေးဌာနခွဲ

(၅) ဖြေဆိုသူ၏နေအိမ် (၆) အခြား (ဖော်ပြရန်) ----------------------------

၅၂။ မီးဖွားပြီးဘယ်လောက်အကြာမှာ စောင့်ရှောက်မှုစ၍ ခံယူခဲ့သည်။

(၁) မီးဖွားပြီး (၂၄)နာရီအတွင်း (၂) (၂၄)နာရီမှ (၄၈)နာရီအတွင်း

(၃) (၄၈)နာရီမှ (၇၂)နာရီအတွင်း (၄) (၇၂)နာရီကြာပြီးနောက်

(၅) တစ်ပတ်ကြာပြီးနောက်

၅၃။ မီးဖွားပြီး (၄၂) ရက်အတွင်း ကျန်းမာရေးစောင့်ရှောက်မှု ဘယ်နှစ်ကြိမ် ခံယူခဲ့သလဲ။--------------------

၅၄။ မီးဖွားပြီးချိန် မီးတွင်းကာလအတွင်း ကျန်းမာရေးစောင့်ရှောက်မှုကို ဘယ်သူ့ဆီက ခံယူခဲ့သလဲ။

(၁) ဆရာဝန် (၂) သူနာပြုဆရာမ (၃) အမျိုးသမီးကျန်းမာရေးဆရာမ

(၄) သားဖွားဆရာမ (၅) အရန်သားဖွား (၆) အရပ်လက်သည်

(၇) အခြား (ဖော်ပြရန်) --------------------

၅၅။ မီးဖွားပြီးချိန် ဘယ်လိုကျန်းမာရေးစောင့်ရှောက်မှုတွေ့ရှိခဲ့ပါသလဲ။

(အဖြေများကို ဖော်ပြရန်လိုသည်။) (တစ်ခုထက်ပို၍ ဖြေဆိုနိုင်ပါသည်။)

(၁) မွေးလမ်းကြောင်းဒဏ်ရာစစ်ခြင်း ၊သားမြတ်စစ်ဆေးခြင်း

(၂) မိခင်နို့တိုက်ကျွေးရေးပညာပေးခြင်း

(၃) သန္ဓေသား ဆေးနည်းများရှင်းလင်းပညာပေးခြင်း

(၄) လိုအပ်သည့် သန္ဓေတားဆေးပေးခြင်း

(၅) မွေးကင်းစကလေးငယ်၏ ကျန်းမာရေးကို စစ်ဆေးပေးခြင်း

(၆) မီးဖွားပြီးချိန် သတိပြုရမည့်အန္တရာယ်များ ကိုရှင်းလင်းပြောပြခြင်း

(၇) VitB ၊ သံဓါတ်ပါသော အားဆေးများပေးခြင်း

၅၆။ မီးဖွားပြီးချိန်နောက်ဆက်တွဲ ဆိုးကျိုး၊ အန္တရာယ်များနှင့် ကြုံတွေ့ခဲ့ရပါသလား။

(၁) ကြုံတွေ့ခဲ့သည်။ (၂) မတွေ့ခဲ့ပါ။

၅၇။ ကြုံတွေ့ခဲ့ရလျှင် မည်သည့်အန္တရာယ် /ပြဿနာနှင့် ကြုံတွေ့ခဲ့ရသနည်း။

(၁) မီးတွင်းသွေးသွန်ခြင်း /သွေးဆင်းများခြင်း

(၂) အပြင်းဖျားခြင်း

(၃) အနာပိုးဝင်ခြင်း

(၄) သားမြတ်ရောင်ခြင်း/ပြည်တည်ခြင်း

(၅) အခြား (ဖော်ပြရန်)------------

၅၈။ မီးဖွားပြီးချိန်မီးတွင်းကာလမှာ အစားအသောက်ရှောင်ခြင်းမျိုး(သို့) အနေအထိုင်၊ အပြုအမူ ကန့်သတ်ဆင်ခြင်ခြင်း မျိုးရှိပါသလား။

(၁) အစားအသောက်ရှောင်ခြင်းရှိသည်။ (ဖော်ပြရန်) --

(၂) အနေအထိုင်၊အပြုအမူကန့်သတ်ဆင်ခြင်ခြင်းရှိသည်။ (ဖော်ပြရန်) -------------------------

(၃) နှစ်မျိုးစလုံး မရှိပါ။
