# Supplementary material for: Utilization of full postnatal care services among rural Myanmar women and its determinants: a cross-sectional study
Source: F1000Res. 2018 Aug 1;7:1167. [Version 1] doi: 10.12688/f1000research.15561.1 (PMC6085599; doi:10.12688/f1000research.15561.1)
Supplement: Peer review history

# Peer review: Referee response for version 1

**DOI:** 10.5256/f1000research.16974.r36686

**Published:** 2018-08-13

**Authors:** Htin Zaw Soe

**Affiliations:** 1 University of Community Health, Magway (UCH), Magway, Myanmar

The work is clearly and accurately presented with current literature. The study design is appropriate. The work is found to be reproducible. Statistical methods are suitable and interpretations are appropriate. Conclusions are supported by results.

 In addition to these points, reviewer’s comments are provided as follow.

**Methods:**

1. Study participants: Add ‘a’ in the sentence of ……….by applying a multistage cluster sampling method.
2. Assessment of variables: Author uses interchangeably the words – ‘explanatory’ and ‘independent’, both of which have same meaning. To avoid reader’s confusion, the author should use only one word – either ‘explanatory’ or ‘independent’. Or use like ….independent (explanatory) variable and then continuously use ‘independent’ only throughout the text.
3. Data analysis: In description of p values, in the text, small letter ‘p’ is used, and in the tables (3) and (4), capital letter ‘P’ is used. Author should use only one type either small or capital to have consistency throughout the text. The small one is better

**Results:**

1. Author uses the words – ‘spouse’ in the text and ‘husband’ in the table. Should use only one type.
2. Table (1): Table construction should be like this. 
3. In Table (2) heading, ‘Factors related to’ is used and in the text ‘Factors relating to’ is used. Use only one type.
4. Table (2): Table construction should be like this.
5. Table (3) and (4): It is better if author constructs two columns separately for – ‘Variables’ - and ‘category’ as suggested above.

**  Discussion:**

1. The prevalence of full PNC utilization should be described with 95% CI, rather than 25.2%.
2. The word ‘our’ should not be used. Use ‘the present study’ instead of ‘our study’.
3. Add ‘be’ in the sentence of …..The possible explanation might be that low income…..
4. Add ‘were’ in the sentence of …..their knowledge about maternal health were significantly related……..
5. In the last sentence of Discussion, one related reference should be added to support the statement of ‘ a 2 year recall period was selected to minimize this bias’.

**Conclusion:**

1.  The determinants should not be described again because they have been already described before.
2. In the last sentence of Conclusion, use ‘is’ instead of ‘should be’, reflecting an        author’s strong suggestion.

 

I have read this submission. I believe that I have an appropriate level of expertise to confirm that it is of an acceptable scientific standard.

# Peer review: Referee response for version 1

**DOI:** 10.5256/f1000research.16974.r36684

**Published:** 2018-08-08

**Authors:** Win Myint Oo

**Affiliations:** 1 Faculty of Medicine, SEGi University, Petaling Jaya, Malaysia

Page 3(Methods)

 You should describe the number of selected villages included in this study. Actually it was 21; isn’t it

 Page 3 (Study Participants) How did you get the sample size “500”; by using formula or soft ware? You better describe the sample size calculation in details.

 What do you mean that the sample size was estimated to be 500, based on the multiple logistic regression analysis, as described previously? Where and how you describe it? I cannot find it at anywhere!

 And you have selected four (4) from 26 townships and five (5) villages from each selected township. It made the number of villages included in your study to be (20). However, the actual number of villages included was (21). It is better to explain why it becomes 21, rather than 20.

 Page 3 (Assessment of Variables)

 Do we need to describe with/from whom the respondents received (or) sought PN care; either MW (mid-wife) or LHV (lady health visitor) in the operational definition of “utilization of full PNC? This is just my suggestion. You can decide not to describe!

 There are three types of determinants/factors associated with the outcome variable ( ***independent variables*** such as age, education, average monthly per-capital income, male involvement and accessibility to PNC service, ***explanatory variables*** such as knowledge of post-natal danger signs and perception on TBAs, and ***important independent variables*** such as birth order, number of AN visit and misconception regarding post-natal practices) in your study! Why do you classify these into three categories? Based on what (or) Why? Is it possible to describe simply (for example, the variables considered as determinants of utilizing full PNC in this study were ------ [or] the present study considered -------- as independent variables)? Or main independent variables were ---- in this study. Those variables ------ were considered as confounders in the present study

 Please try to be consistent in utilizing the name of variables. (At first you use the name of independent variable as **accessibility to PNC service** but later you use its name as **accessibility to the [nearest] health center**. Accessibility to the nearest health center seems to be more relevant. Another one is concerned with **perception on TBA**. Although you use the term **perception**, later you changed it to **acceptance**. I think “acceptance” is more appropriate.

 Page 6 (Discussion)

 Please insert the in-text citation for the description of (less than 50,000 MMK; the amount below the international poverty line as determined by the World Bank).

 Page 8&9 (Conclusion)

 Based on the findings of your study, maternal education level/status was found to be significantly associated with utilization of full PNC. Therefore, you better add the statement/conclusion regarding with that variable into your conclusion! (For example, maternal education or education of women especially in rural area should be enhanced/improved in order to promote the utilization of full PNC or something like that).

I have read this submission. I believe that I have an appropriate level of expertise to confirm that it is of an acceptable scientific standard.

# Peer review: Referee response for version 1

**DOI:** 10.5256/f1000research.16974.r36685

**Published:** 2018-08-06

**Authors:** Myo Myo Mon

**Affiliations:** 1 Department of Medical Research, Ministry of Health and Sports, Yangon, Myanmar

It should be accepted.

 It’s a good article highlighting the determinant of utilization of PNC services among rural women in Myanmar.

 The work is clearly and accurately presented. The finding clearly fulfilled the objectives of the study. And it also includes the available current literature.

 The study design is appropriate and the work is technically sound. Investigators used the sound study design, measured all necessary outcome and independent variables, and defining all the operational definitions. There are sufficient details of methods and analysis provided to allow replication by others. The statistical analysis and its interpretation are appropriate. Statistical methods are clearly described and used the correct method of analysis to fulfill the objectives. All the source data underlying the results are available to ensure full reproducibility. The conclusions drawn are adequately supported by the results.

**General comments**

 Please describe one or two sentence about pre-test if possible. If validity was ensured by obtaining the three experts’ comments, is it possible to show content validity index? If not, it’s OK.

 Please move the description about the categorization of level of awareness of postnatal danger signs under the findings section to include under the “assessment of variables”.  Similar comment is for the definition of skilled care provider.

I have read this submission. I believe that I have an appropriate level of expertise to confirm that it is of an acceptable scientific standard.

# Response

**Published:** 2018-08-07

**Authors:** Aye Sandar Mon

**Affiliations:** Khon Kaen University, Thailand

We would like to express our sincere gratitude and appreciation to Dr. Myo Myo Mon for your effort on this manuscript.

 Regarding the pretest, 25 reproductive-aged mothers who had under 2-years aged children from the selected rural area of Yangon Region were interviewed using same questionnaire that was constructed based on WHO Recommended Interventions for Improving Maternal and Newborn Health, WHO Guidelines Approved by the Guidelines Review Committee: Pregnancy, Childbirth, Postpartum and Newborn Care: A Guide for Essential Practice and Myanmar multiple indicator cluster survey.

 We are truly sorry that we cannot mention the content validity index.

 We agree your suggestion to add the operational definition and categorization of the variables, level of awareness on postnatal danger signs and type of maternal health care providers, under the subheading “Assessment of variables”.
